# Downregulation of deoxycytidine kinase in cytarabine-resistant mantle cell lymphoma cells confers cross-resistance to nucleoside analogs gemcitabine, fludarabine and cladribine, but not to other classes of anti-lymphoma agents

**DOI:** 10.1186/1476-4598-13-159

**Published:** 2014-06-27

**Authors:** Magdalena Klanova, Lucie Lorkova, Ondrej Vit, Bokang Maswabi, Jan Molinsky, Jana Pospisilova, Petra Vockova, Cory Mavis, Lucie Lateckova, Vojtech Kulvait, Dana Vejmelkova, Radek Jaksa, Francisco Hernandez, Marek Trneny, Martin Vokurka, Jiri Petrak, Pavel Klener

**Affiliations:** 1 Institute of Pathological Physiology, Charles University in Prague, First Faculty of Medicine, Prague, Czech Republic; 2 First Department of Medicine - Department of Hematology, General University Hospital and Charles University in Prague, Prague, Czech Republic; 3 Institute of Pathology, General University Hospital and Charles University in Prague, Prague, Czech Republic; 4 Departments of Immunology and Medicine, Roswell Park Cancer Institute, Buffalo, NY, USA; 5 Institute of Hematology and Blood Transfusion, Prague, Czech Republic

**Keywords:** Mantle cell lymphoma (MCL), Cytarabine, Drug resistance, Nucleotide salvage pathway, Proteomics, Mass spectrometry

## Abstract

**Background:**

Mantle cell lymphoma (MCL) is an aggressive type of B-cell non-Hodgkin lymphoma associated with poor prognosis. Implementation of high-dose cytarabine (araC) into induction therapy became standard-of-care for all newly diagnosed younger MCL patients. However, many patients relapse even after araC-based regimen. Molecular mechanisms responsible for araC resistance in MCL are unknown and optimal treatment strategy for relapsed/refractory MCL patients remains elusive.

**Methods:**

Five araC-resistant (R) clones were derived by long-term culture of five MCL cell lines (CTRL) with increasing doses of araC up to 50 microM. Illumina BeadChip and 2-DE proteomic analysis were used to identify gene and protein expression changes associated with araC resistance in MCL. *In vitro* cytotoxicity assays and experimental therapy of MCL xenografts in immunodeficient mice were used to analyze their relative responsiveness to a set of clinically used anti-MCL drugs. Primary MCL samples were obtained from patients at diagnosis and after failure of araC-based therapies.

**Results:**

Marked downregulation of deoxycytidine-kinase (DCK) mRNA and protein expression was identified as the single most important molecular event associated with araC-resistance in all tested MCL cell lines and in 50% primary MCL samples. All R clones were highly (20-1000x) cross-resistant to all tested nucleoside analogs including gemcitabine, fludarabine and cladribine. *In vitro* sensitivity of R clones to other classes of clinically used anti-MCL agents including genotoxic drugs (cisplatin, doxorubicin, bendamustine) and targeted agents (bortezomib, temsirolimus, rituximab) remained unaffected, or was even increased (ibrutinib). Experimental therapy of immunodeficient mice confirmed the anticipated loss of anti-tumor activity (as determined by overall survival) of the nucleoside analogs gemcitabine and fludarabine in mice transplanted with R clone compared to mice transplanted with CTRL cells, while the anti-tumor activity of cisplatin, temsirolimus, bortezomib, bendamustine, cyclophosphamide and rituximab remained comparable between the two cohorts.

**Conclusions:**

Acquired resistance of MCL cells to araC is associated with downregulation of DCK, enzyme of the nucleotide salvage pathway responsible for the first phosphorylation (=activation) of most nucleoside analogs used in anti-cancer therapy. The data suggest that nucleoside analogs should not be used in the therapy of MCL patients, who relapse after failure of araC-based therapies.

## Background

Mantle cell lymphoma (MCL) is an aggressive type of B-cell non-Hodgkin lymphoma (NHL) associated with poor prognosis [1,2]. In recent years several studies brought evidence that implementation of high-dose cytarabine (araC) into induction therapy, e.g. by sequential chemotherapy by R(ituximab)-CHOP and R-DHAP regimens, induced higher response rate and prolonged progression-free survival compared to R-CHOP-only [3-5]. Based on these results, implementation of araC into induction therapy became standard of care for all newly diagnosed younger MCL patients. Despite considerable improvement, however, most high-risk MCL patients relapse even after araC-based first-line regimen. Prognosis of relapsed/refractory (RR) MCL is dismal. Currently, there is no second-line standard-of-care for RR-MCL [6]. Available treatment approaches for RR-MCL include cisplatin, fludarabine, cladribine, gemcitabine, temsirolimus, bortezomib, bendamustine, lenalidomide and ibrutinib-based regimen [7-16].

AraC belongs among the backbone anti-leukemia agents [17]. Both, “standard dose” araC (100-200 mg/m2 continuous i.v. infusion for 7 days), and “high dose” araC (HDAC, 2-3 g/m2, 2–4 i.v. three hour administrations every 12–24 hours) have been widely used in the therapy of acute myelogenous leukemia (AML), as well as in salvage regimen for relapsed B-NHL [18,19]. As mentioned above araC appears particularly effective component of multi-agent aggressive immunochemoterapy regimen used in younger MCL patients.

AraC is a prodrug, which must be 1. transported into the cell, and 2. within the cell converted into an active drug by phosphorylation by specific phosphokinases of the nucleotide salvage pathway [20]. During “standard dose” cytarabine administration araC is transported into the cell by means of specific transporters, primarily via hENT1/SLC29A1 [21]. During high-dose cytarabine administration araC also diffuses across plasma membrane independent of the specific transporters [22]. The rate-limiting enzyme of the nucleotide salvage pathway is deoxycytidine-kinase (DCK), which catalyzes the first phosphorylation of araC into araCMP. AraCMP is retained in the cell and undergoes two additional consecutive phosporylations before it can be incorporated into DNA.

The molecular mechanisms of araC resistance in MCL are unknown. Resistance to araC in myeloid leukemia cells was repeatedly associated with altered expression of genes involved in nucleotide salvage pathway, including downregulation of DCK, or upregulation of key araC-inactivating enzymes, namely cytidine-deaminase (CDA) or cytoplasmic 5′nucleotidase (NT5C2) [20-25].

In this study we derived araC-resistant MCL cells, studied their sensitivity to a battery of anti-cancer drugs and elucidated the molecular mechanism responsible for araC resistance in MCL.

## Results

### Establishment and characterization of araC-resistant MCL clones (R clones)

**Figure 1:**
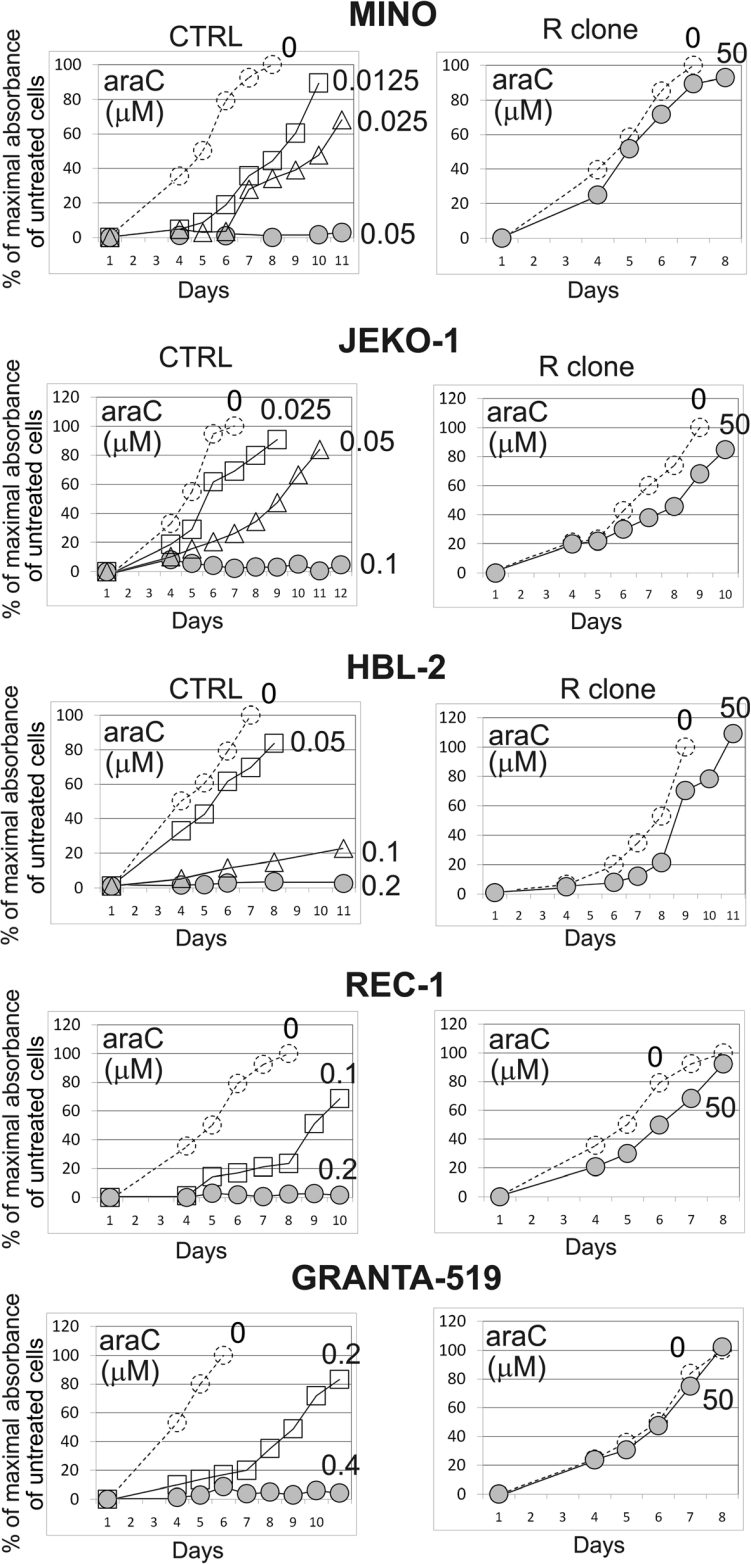
**R clones are resistant to 50 μM cytarabine.** WST-8 cell proliferation assay of 5 MCL cell lines (CTRL) and 5 R clones was carried out as described in Methods. While the lethal dose of cytarabine for CTRL cells ranged from 0.05 to 0.4 μM, proliferation rate of R clones in 50 μM araC was virtually unaffected. Representative example of two independent experiments is shown. Standard deviations were < 5% for all measurements.

Five araC-resistant MCL clones (=R clones) were established by long-term culture of five cytarabine-sensitive MCL cell lines (JEKO-1, MINO, REC-1, HBL-2 and GRANTA-519, =CTRL cell lines) in the presence of increasing doses of araC (up to 50 μM, comparable with plasma concentration reached in patients treated with high-dose araC) [26]. Resistance of R clones to araC was confirmed *in vitro* by proliferation assays (Figure 1). The R clones tolerated at least 125-1000-fold higher concentrations of araC compared to CTRL cells (Figure 1).

### Gene expression profiling of R clones revealed downregulation of deoxycytidine-kinase (DCK)

**Figure 2:**
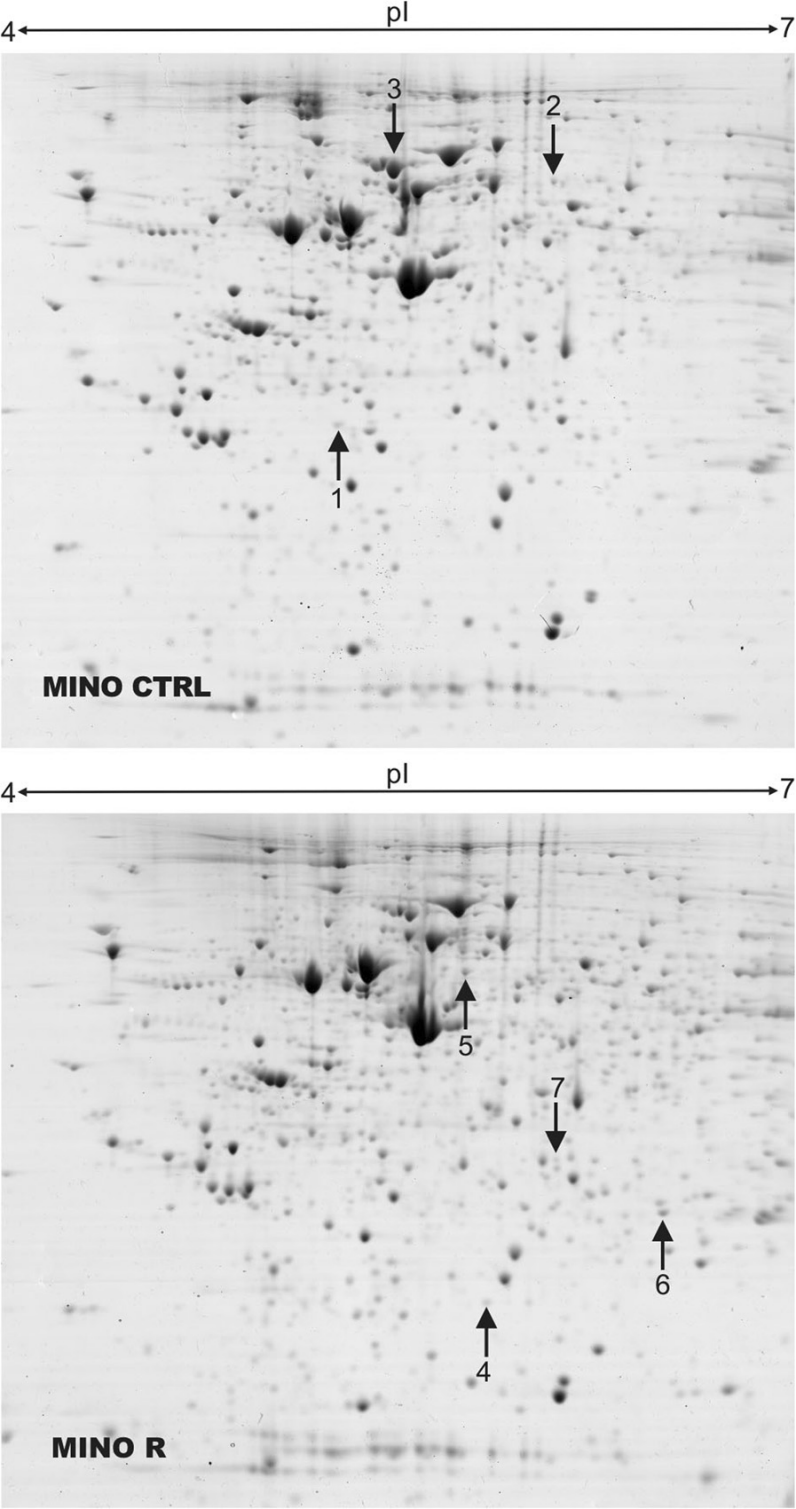
**Proteomic analysis of MINO R vs MINO CTRL cells.** Two-dimensional electrophoresis of cells MINO R cell versus MINO CTRL cells was performed on 24 cm gel strips, pH 4.0-7.0, 10% SDS-PAGE. Proteins were stained with Coomassie Brilliant Blue. Differentially expressed proteins are indicated by numbered arrows, spots 1–3 indicate proteins significantly downregulated in MINO R cells, and spots 4–7 indicate proteins upregulated in MINO R cells.

**Figure 3:**
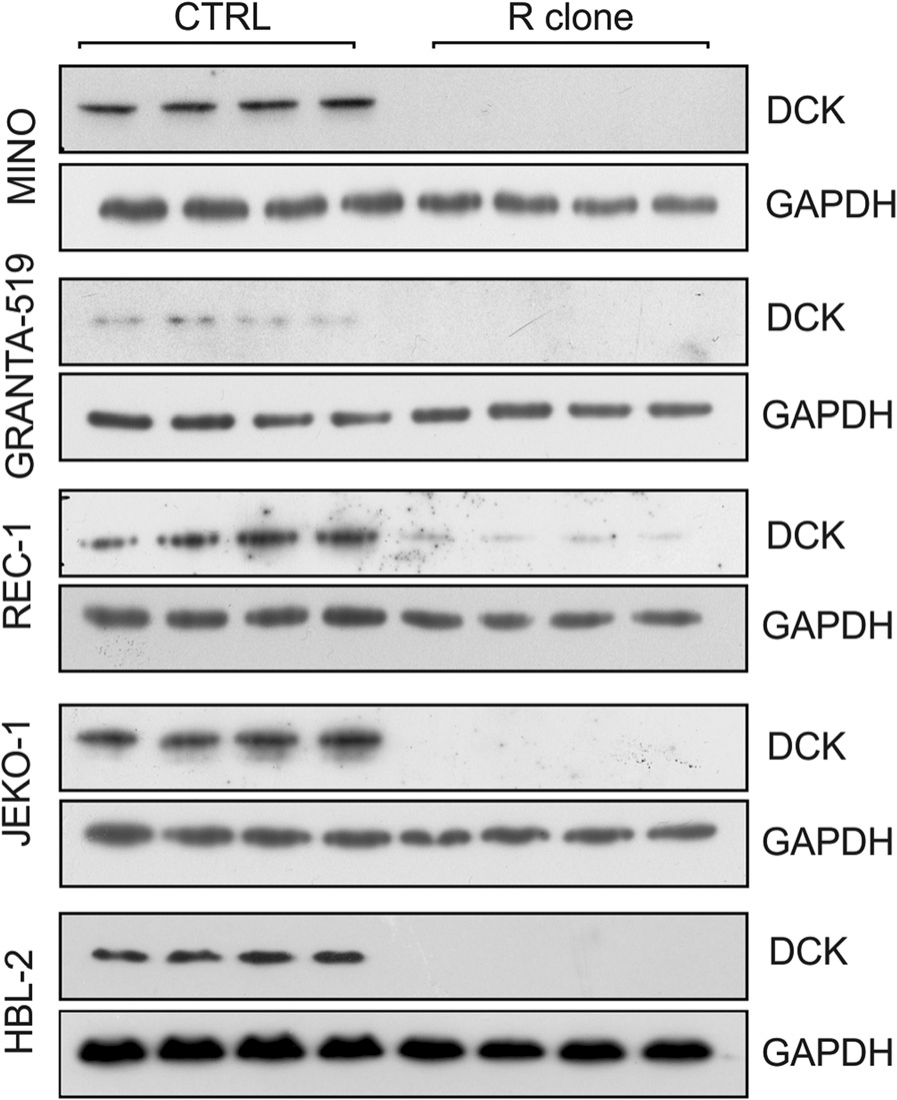
**Western blot analysis confirms marked downregulation of protein DCK in all R clones.** Relative expression of deoxycytine kinase (DCK) in all five R and CTRL clones. Quadruplicate cell lysates were separated on 12% SDS-PAGE minigels. Proteins were then transferred onto PVDF membranes, blocked and probed with anti-DCK antibody. Anti-GAPDH antibody was used as the loading control.

To identify gene and protein expression changes associated with araC resistance in MCL we performed parallel transcriptome profiling and proteomic analysis of R clones compared to CTRL cell lines. Transcriptomic analysis was performed for each of the 5 MCL cell lines and their respective R clones in biological duplicates using Illumina BeadChips. The filtered groups of genes with fold change at least ± 1.5-fold and adjusted p value < 0.05 were annotated and arranged into biologically relevant categories using The Database for Annotation, Visualization and Integrated Discovery (DAVID, Additional file 1: Figure S1). Based on Gene Ontology (GO) terms, the downregulated genes were involved in *ribosome structure and function*, *cell cycle*, *RNA degradation*, *antigen processing and presentation*, *purine metabolism* and *pyrimidine metabolism* (Additional file 1: Figure S1A). Among the most upregulated gene groups belonged those involved in *graft-vs-host disease*, *alograft rejection*, *B-cell receptor signaling*, *cell adhesion molecules*, *chemokine signaling pathway* and *Toll-like receptor signaling* (Additional file 1: Figure S1B). The only gene consistently differentially expressed across all 5 MCL cell lines was DCK, which was markedly downregulated in all R clones. Other genes differentially expressed in more than one MCL cell line are shown in Additional file 2: Table S1. Proteomic analysis using 2-DE was applied to Mino R subclone compared to Mino CTRL cell line, and revealed differential expression of several proteins, among them almost 5-fold downregulation of DCK in the Mino R subclone was the most apparent (Figure 2, Tables 1 and 2). Downregulation of DCK protein (the rate-limiting enzyme of the nucleotide salvage pathway, which catalyzes the first phosphorylation of araC and other nuclosides into their respective monophosphates) was confirmed by western blotting in all five R clones (Figure 3). DCK expression seemed to be fully abrogated in four R clones (as there was no detectable DCK) and several-fold downregulated in one R clone compared to the CTRL cells.

**Table 1:** List of proteins differentially expressed in MINO R cells identified by 2-DE

**Spot no.**	**Accession**	**Protein name**	**Fold change**	**Mascot score**	**Sequence cov. (%)**	**Mr**
**Proteins downregulated in MINO R cells**						
1	P27707	Deoxycytidine kinase	−4.6	44^*^	16	30841
2	Q99829	Copine-1	−4.3	102	17	59649
3	P13796	Plastin-2	−2	453	65	70814
**Proteins upregulated in MINO R cells**						
4	P07741	Adenine phosphoribosyltransferase	5	70	40	19766
5	P68363	Tubulin alpha-1B chain	5	169	32	50804
6	P04792	Heat shock protein beta-1	2/3	73	32	22826
7	P31937	3-Hydroxyisobutyrate Dehydrogenase, Mitochondrial	2/1	43*	8	35712

*Identity of proteins with low Mascot Score was verified by MS/MS (see Table 2).

Included are the proteins with difference in expression at least 2-fold and statistical significance of the change p < 0.05. Swiss-Prot no. is the code under which the identified protein is deposited in the Swiss-Prot database. Mascot score helps to estimate the correctness of the individual hit. It is expressed as −10 × log(P) where P is the probability that the observed match is a random event. Sequence coverage is the number of amino acids spanned by the assigned peptides divided by the sequence length.

**Table 2:** Identity of differentially expressed proteins with low mascot score confirmed by MS/MS

**Spot no.**	**Accession**	**Protein name**	**Peptide sequence**	**Score**
1	P27707	Deoxycytidine kinase	LKDAEKPVLFFER, QLCEDWEVVPEPVAR	41, 46
7	P31937	3-Hydroxyisobutyrate Dehydrogenase, Mitochondrial	DFSSVFQFLREEETF (C-term), SPILLGSLAHQIYR	49, 28

### AraC-resistant clones are cross-resistant to nucleoside analogs, but remain sensitive to other classes of anti-lymphoma agents

**Figure 4:**
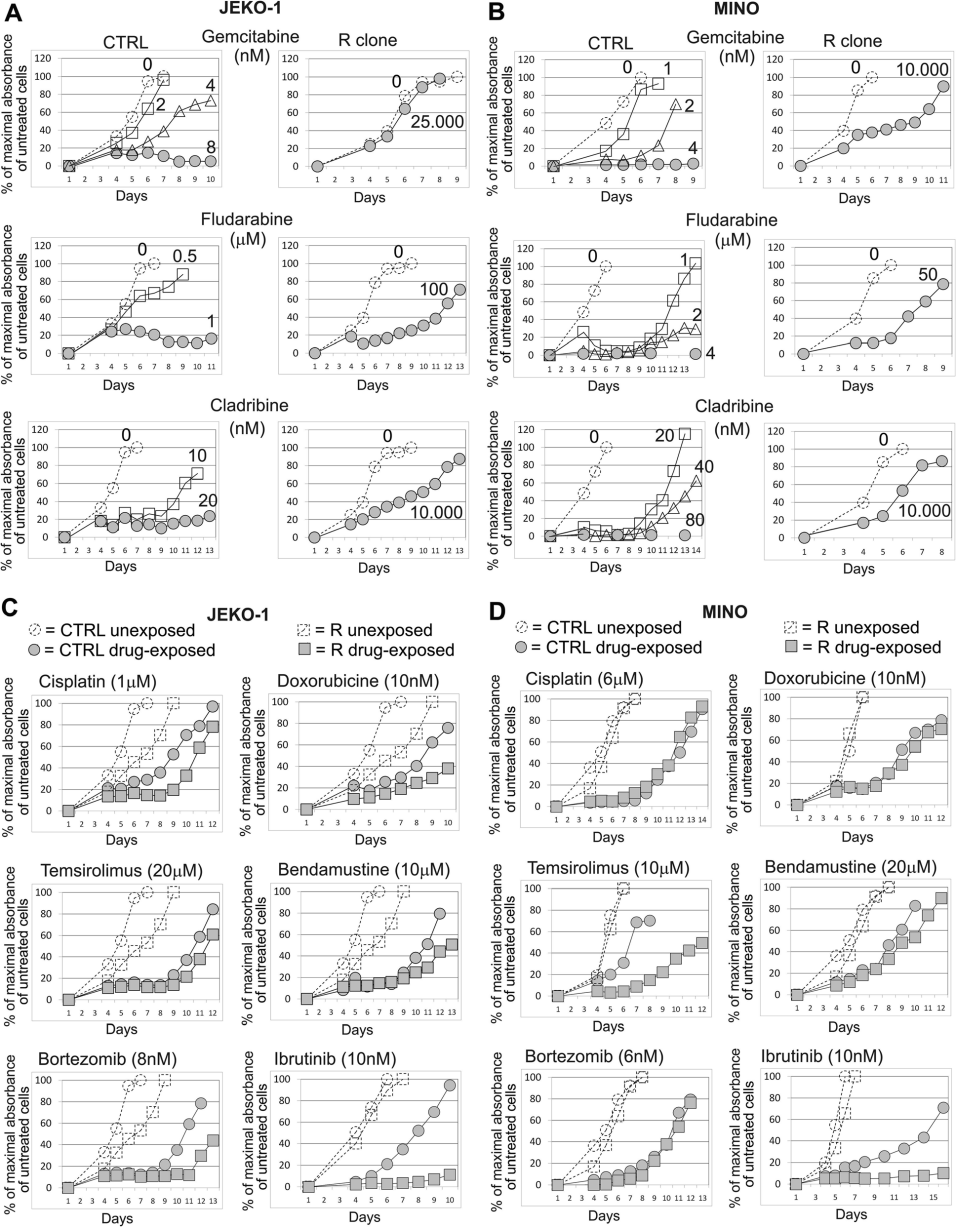
**R clones are cross-resistant to nucleoside analogs, but remain sensitive to other classes of anti-lymphoma agents. (A**-**D)** WST-8 cell proliferation assays of CTRL cells and R clones were carried out as described in Methods. Maximal absorbance obtained from the untreated cells during the particular experiment (MAX_u_) was arbitrary set as 100%. Absorbance of medium without cells was used as background **(B)**. For each cell population (both, unexposed and drug-exposed) and for each measurement (M_1_, M_2_, M_3_…M_X_) the proliferation curve was calculated as follows: (M_X_ - B)/(MAX_u_ - B). As a consequence, proliferation curves of untreated cells always peak at 100%, while proliferation curves of drug-exposed cells can terminate below or above 100%. One representative example of two independent experiments carried out both on JEKO-1 **(A, C)** and MINO **(B, D)** is shown. Data from the remaining three MCL cell lines (HBL-2, GRANTA-519 and REC-1) are not shown, because they did not significantly differ from those presented for the JEKO-1 and MINO cells. In summary, all 5 R clones were cross-resistant to the tested nucleoside analogs, but remained sensitive to other classes of anti-lymphoma agents with negligible differences between particular MCL cell lines. The only exception to the rule was markedly (>100-fold) increased sensitivity of REC-1 R clone to ibrutinib compared to REC-1 CTRL cells (see Additional file 3: Figure S2). The remaining 4 MCL cell lines (JEKO-1, MINO, GRANTA-519 and HBL-2) showed only approx. 2-fold increased sensitivity to ibrutinib compared to the corresponding CTRL cells. Standard deviations were < 5% for all measurements presented in Figure 4.

**Figure 5:**
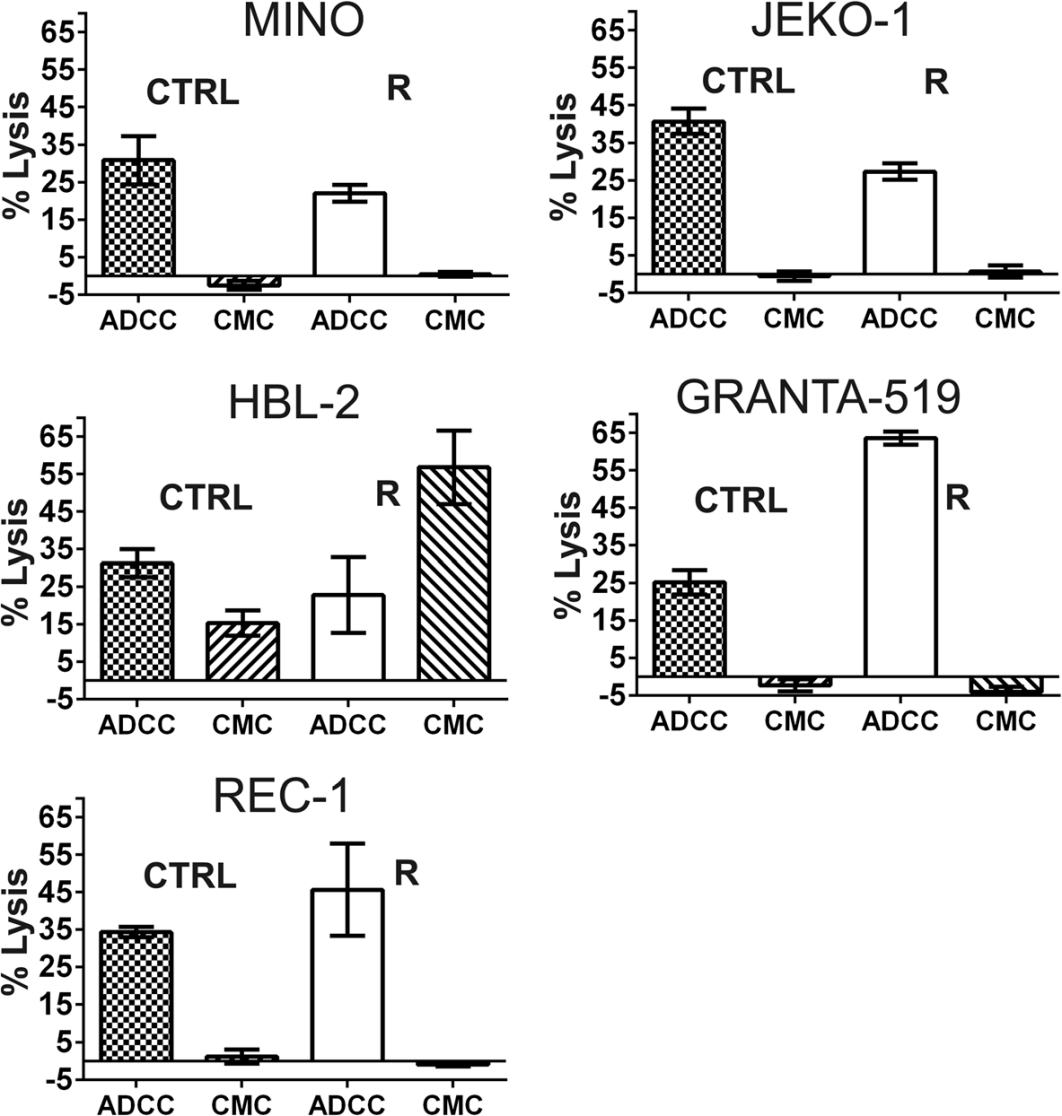
**R clones remain sensitive to anti-CD20 monoclonal antibody rituximab. **^51^Cr release assay was used to assess the impact of the anti-CD20 monoclonal antibody rituximab on complement mediated cytotoxicity (CMC) and antibody dependent cellular cytotoxicity (ADCC). CMC was measurable only in HBL-2 cells (both CTRL and R), but was negligible in the remaining four MCL cell populations (both CTRL and R). In HBL-2 R clone the CMC was significantly increased compared to CTRL. ADCC was measurable in all five MCL cell lines. In JEKO-1 R clone the ADCC was slightly decreased compared to CTRL. In GRANTA-519 R clone the ADCC was significantly increased compared to CTRL. In MINO, REC-1 and HBL-2 the ADCC remained comparable between R clone and CTRL cells.

To identify optimal treatment strategy for araC-resistant MCL we determined sensitivity (or eventual cross-resistance) of all 5 R clones in a battery of cellular toxicity tests. We exposed R clones and CTRL cells to a panel of clinically used anti-MCL agents in various concentrations and measured their effect on cell proliferation rate. The tested agents included both, classical genotoxic cytostatics and novel targeted drugs. The panel included alkylating agents cisplatin, doxorubicin and bendamustine, nucleoside analogs gemcitabine, cladribine and fludarbine, and targeted drugs bortezomib (proteasome inhibitor), temsirolimus (mTOR inhibitor) and ibrutinib (Bruton tyrosine-kinase (BTK) inhibitor). All five R clones (resistant to a pyrimidine analog cytarabine) showed cross-resistance not only to another pyrimidine analog gemcitabine (up to 3125-fold), but also to purine nucleoside analogs fludarabine and cladribine (approx. 12.5-500-fold, see Figure 4A,B). Sensitivity of the resistant R clones to other classes of anti-lymphoma agents (i.e. other than nucleoside analogs) remained comparable to the respective CTRL cells (Figure 4C,D), with the exception of ibrutinib. The BTK inhibitor ibrutinib proved to be significantly more cytotoxic to R clones compared to CTRL cells *in vitro* (see Figure 4C,D, Additional file 3: Figure S2). R clones also retained *in vitro* sensitivity to anti-CD20 monoclonal antibody rituximab comparable to CTRL cells as determined by ^51^Cr release assay, which is standardly used to evaluate antibody-dependent cytotoxicity (ADCC) and complement-mediated cytotoxicity (CMC) of therapeutic monoclonal antibodies (Figure 5).

### Experimental therapy with fludarabine and gemcitabine is ineffective in mice xenografted with araC-resistant clones

**Figure 6:**
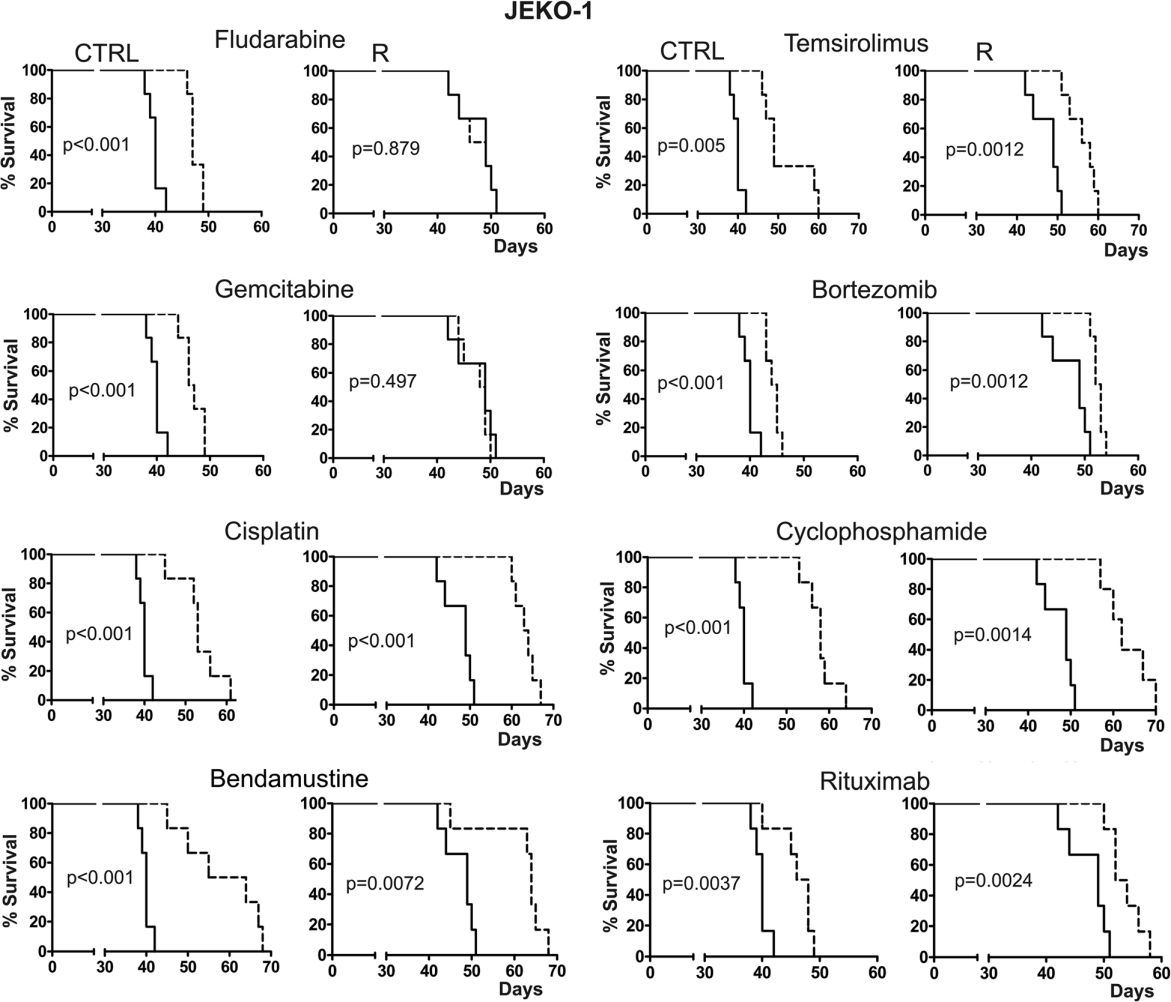
**Experimental therapy of mice xenografted with JEKO-1 CTRL and JEKO-1 R cells.** Overall survival of experimental animals is shown as Kaplan-Meier survival estimates. Loss of anti-tumor activity was observed for the nucleoside analogs fludarabine and gemcitabine in the therapy of R clone-xenografted mice (dashed lines) when compared to CTRL-xenografted mice (solid lines). Other classes of anti-lymphoma agents retained the antitumor activity in both CTRL- and R clone-xenografted mice. Individual cohorts contained 6 animals. For more details see Methods.

The *in vitro* tests of cellular toxicity provided important information on direct cellular effects of the tested drugs to the resistant cells. However, *in vitro* assays do not take into account important systemic pharmacokinetic and pharmacodynamic variables, which can have large impact on the drug efficacy *in vivo*. In addition, some anti-MCL agents cannot be properly tested *in vitro*, because their mechanism of antitumor activity directly or indirectly depends on the *in vivo* context, e.g. activation of a prodrug cyclophosphamide in the liver microsome, cooperation of a monoclonal antibody rituximab with complement and cells of the immune system, or antiangiogenic component of temsirolimus activity. Therefore, we used a mouse xenograft model (NOD.Cg-*Prkdc*^*scid*^*Il2rg*^*tm1Wjl*^/SzJ mice) of MCL to simulate *in vivo* treatment of araC-sensitive and araC-resistant disease. Intravenous injection of 1 million JEKO-1 MCL cells leads to demise of the xenografted animals due to disseminated lymphoma with median overall survival of approx. 38 days. Experimental therapy of JEKO-1-xenografted immunodeficient mice with single-agent fludarabine and gemcitabine confirmed total loss of anti-tumor activity of purine analog fludarabine and pyrimidine analog gemcitabine (measured as overall survival of experimental animals) in mice transplanted with cytarabine-resistant JEKO-1 R clone compared to mice transplanted with cytarabine-sensitive JEKO-1 CTRL cells (Figure 6). Anti-tumor activity of cisplatin, temsirolimus, bendamustine, bortezomib, cyclophosphamide and rituximab remained comparable between JEKO-1 R clone and JEKO-1 CTRL-xenografted mice in agreement with the *in vitro* tests (Figure 6).

### Analysis of primary MCL samples confirmed that downregulation of DCK is frequently associated with failure of high-dose araC-based treatments

**Figure 7:**
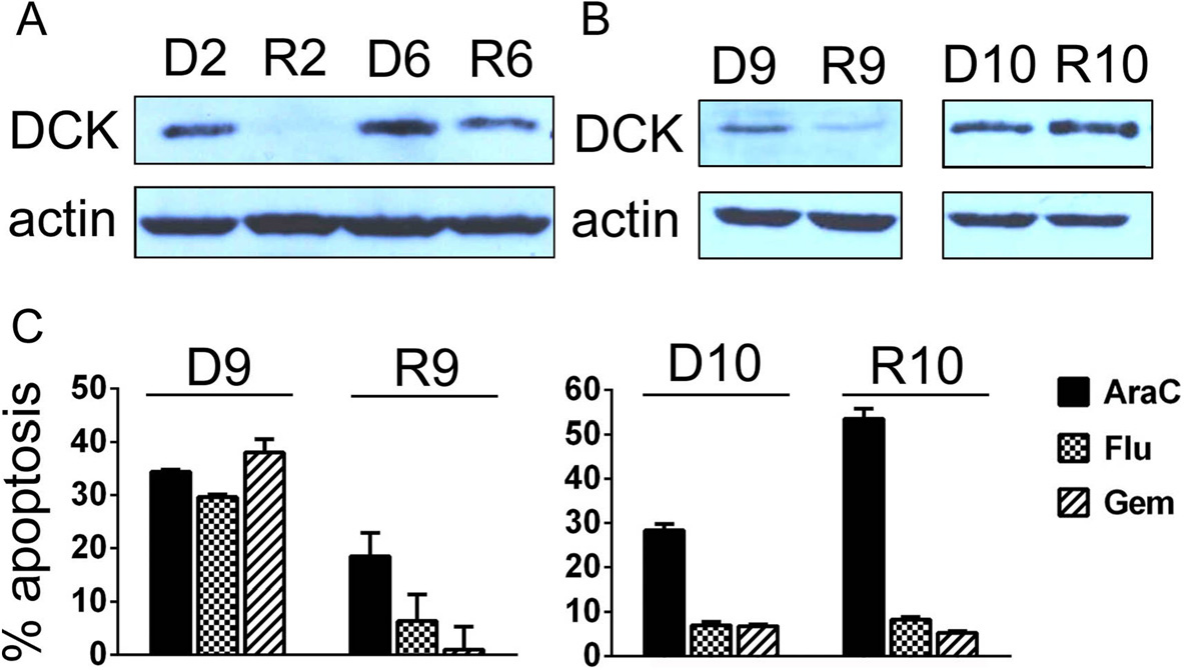
**Protein expression of DCK in primary MCL samples, and their *ex vivo *sensitivity to nucleoside analogs. (A**-**B)** Relative expression of deoxycytine kinase (DCK) in post-treatment primary MCL samples (R2, R6, R9, R10) compared to pre-treatment samples (D2, D6, D9, D10). **(C)** CD19-sorted PBMC cells were isolated from two leukemized araC-refractory MCL patients before (D9, D10) and 14 days after (R9, R10) administration of high-dose araC. The cells were *ex vivo/in vitro* exposed to araC (25 μM), fludarabine (Flu, 100 μM) and gemcitabine (Gem, 25 μM). Apoptosis was measured after 24 hours using standard Annexin-V-PE and flow cytometry. Apoptotic cells are shown as Annexin-V-PE-positive cells (Y axis). Basal apoptosis of drug-unexposed cells was subtracted from the apoptosis of the drug-exposed cells.

Eight and two primary MCL samples obtained from patients at diagnosis (D1-D8) and at lymphoma relapse after failure of high-dose araC-based treatments (R1-R8) were analyzed by real-time RT-PCR and western blotting, respectively (Table 3, Figure 7A). In four cases downregulation of DCK gene expression was observed in R compared to D samples (difference in ΔCT (DCK-GAPDH) between R and D samples was > 1 cycle), while in four cases no change was observed (difference in ΔCT < 1 cycle) (Table 3). Western blotting analysis of the sample R2 compared to D2 revealed marked downregulation of protein DCK thereby confirming the gene expression results (i.e. 4-fold decrease in total DCK mRNA after araC-based therapy). Interestingly, protein DCK in the sample R6 compared to D6 was also moderately downregulated despite its gene expression remained virtually unchanged (Figure 7A, Table 3). In addition to the analysis of MCL samples obtained from the relapsed patients, paired primary cells isolated from two MCL patients (samples D9/R9, and D10/R10) refractory to araC were subject to analysis of gene and protein expression, and determination of their *ex vivo* sensitivity to nucleoside analogs (Figure 7B,C). The samples were obtained before araC administration (D9, D10), and 14 days after araC administration (R9, R10). Downregulation of both gene and protein DCK expression was confirmed in R9 compared to D9 cells (Figure 7B). Sensitivity of R9 cells to araC, fludarabine and gemcitabine was significantly suppressed compared to D9 cells (Figure 7C). Both gene expression and protein expression of R10 compared to D10 sample remained unchanged (Figure 7B). Interestingly, susceptibility of R10 cells compared to D10 cells to undergo apoptosis after their *ex vivo* exposure to araC was increased despite the fact that R10 cells were isolated after administration of four cycles of high-dose araC (Figure 7C).

**Table 3:** Gene expression analysis of DCK in a set of primary MCL samples obtained from patients before and after araC-based therapies

**Sample at diagnosis**	**Source**	**∆CT (DCK-GAPDH)**	**Therapy**	**Sample at relapse**	**Disease-free survival (months)**	**Source**	**∆CT (DCK-GAPDH)**	**Difference in ∆CT between R and D samples**
D1	PBMC	3.4	A*	R1	12	PBMC	3.7	+0.3
D2	PE***	3.3	A	R2	10	PE***	5.3	+2.0
D3	FFPE	0.1	A	R3	5	FFPE	1.3	+1.2
D4	FFPE	1.7	B	R4	4	FFPE	3.5	+1.8
D5	PBMC	1.4	A	R5	7	PBMC	2.2	+0.8
D6	PBMC	4.1	B**	R6	3	PBMC	3.9	−0.2
D7	FFPE	1.3	B	R7	13	FFPE	3.5	+2.2
D8	FFPE	2.0	A	R8	25	FFPE	1.8	−0.2
D9	PBMC	1.9	B	R9	N/A	PBMC	3.3	+1.4
D10	PBMC	2.3	A	R10	N/A	PBMC	1.5	−0.8

*A = alternation of R-CHOP and R-araC (2 g/m2, 2 doses a 24 h).

**B = Nordic protocol (alternation of R-MaxiCHOP and R-araC (2-3 g/m2, 4 doses a 12 h).

***PE pleural effusion (CD19-sorted).

Samples from relapsed patients were obtained at diagnosis (D1-D8) and at lymphoma relapse after failure of araC-based therapies (R1-R8). Samples from refractory patients were obtained from primary araC-resistant MCL patients before (D9-D10) and 14 days after (R9-R10) administration of high-dose araC. Real-time RT-PCR was used to determine changes in DCK expression.

## Discussion

In this study we analyzed molecular mechanisms of araC resistance in five MCL cell lines and ten paired primary MCL samples obtained before and after araC-based therapies. In addition, we tested optimal treatment strategies for cytarabine-resistant MCL. On molecular level we identified marked and principal downregulation of DCK, the rate-limiting enzyme of nucleotide salvage pathway, in all 5 cytarabine-resistant MCL clones, and in 50% primary MCL samples obtained from patients, who progressed on or relapsed after araC-based treatments. In 50% primary MCL samples, no change of DCK expression was observed at time of lymphoma relapse or progression. Importantly, no upregulation of DCK was observed in any of the analyzed post-treatment samples. Although the analysis of the primary MCL samples indicate that the mechanisms responsible for araC resistance *in vivo* are more complex than those observed *in vitro*, it must be emphasized that downregulation of gene and protein DCK was indeed confirmed in a substantial part of the patients’ post-treatment samples (Table 3, Figure 7A,B). Interestingly, in one of the two MCL patients primary resistant to araC, no change of DCK expression was observed with slightly increased *ex vivo* sensitivity of post-treatment MCL cells to araC (Figure 7B,C). This observation could be explained by existence of araC-resistant stem cell-like MCL cells that would reside in the niches in lymph nodes (and/or bone marrow) and produce partially araC-sensitive MCL cells mobilized in the peripheral blood. In such a case, elimination of the mobilized MCL cells, but persistence of the stem cell-like MCL compartment, would lead to stable disease, and eventual lymphoma progression (which was the actual course of the disease observed in this patient).

DCK catalyzes the first phosphorylation (=activation necessary for their cytotoxic activity) not only of araC into araCMP, but also of most nucleoside analogs (both pyrimidine and purine-derived) commonly used in anti-cancer therapy. Using DAVID bioinformatic analyzer *purine/pyrimidine metabolism*, and *B-cell receptor signaling* were among the functional cathegories associated with the most downregulated and upregulated genes, respectively. In accordance with these results we subsequently showed that all R clones were cross-resistant to both pyrimidine analog gemcitabine, and to purine analogs fludarabine and cladribine (all of which are activated by DCK). Sensitivity of R clones to other types of anti-cancer molecules including genotoxic cytostatics (cisplatin, doxorubicin, bendamustine), targeted drugs (temsirolimus, bortezomib) or biological agents (monoclonal anti-CD20 antibody rituximab) remained unaffected, or was even augmented in the case of BTK inhibitor ibrutinib. The reason, why ibrutinib more effectively eliminated araC-resistant MCL cells remained elusive, but might be at least partially explained by the observed upregulation of B-cell receptor signaling in R clones compared to CTRL cells (Additional file 1: Figure S1).

The results of our *in vitro* and *in vivo* tests combined with the observed decreased expression of DCK in all araC-resistant MCL clones and in 50% post-treatment primary MCL samples suggest that the resistance of MCL cells to high-dose araC is caused by suppressed araC activation by DCK due to markedly decreased DCK expression. DCK has low substrate preference and phosphorylates both, purines and pyrimidines, including synthetic analogs cytarabine, fludarabine, gemcitabine and cladribine [27-29]. The fact that above-mentioned nucleoside analogs are substrates of DCK explains the observed cross-resistance of R clones to all tested nucleoside analogs, both purine- and pyrimidine-derived. Retained sensitivity to other classes of anti-MCL agents (i.e. other than nucleoside analogs) with diverse molecular mechanisms of their respective antitumor activities suggests that no major additional molecular alteration was involved in the development of araC resistance.

Prognosis of patients with relapsed/refractory MCL (RR-MCL) is dismal. Currently there is no standard-of-care for RR-MCL patients. Second-line treatment approaches include fludarabine, gemcitabine, cladribine, cisplatin, bortezomib, temsirolimus, bendamustine, lenalidomide and ibrutinib-based regimen. We have proved *in vitro* and *in vivo* on a mouse xenograft model of MCL that treatment of patients, who progress on or relapse after high-dose araC-based regimen should not rely on nucleoside analogs, namely on the currently used agents fludarabine, gemcitabine and cladribine, since all of them must be phosphorylated by DCK to exert their anti-lymphoma activity. Instead, other classes of anti-lymphoma drugs should be applied in case of araC failure, i.e. in the setting of anticipated araC-resistance. Some of these agents have only recently been approved for the therapy of relapsed/refractory (RR-) MCL, temsirolimus in Europe, bortezomib and ibrutinib in USA. It might be speculated that high-dose therapy (given before autologous stem cell transplant) based on other agents than nucleoside analogs might prove more beneficial especially for those patients with suboptimal responses after induction araC-based immunochemotherapy (e.g. patients, who achieve partial remission, or patients with detectable minimal residual disease). In addition to the currently approved agents, bendamustine represents another extremely promising drug in MCL. Recently it was demonstrated that bendamustine potentiates the effect of araC by augmenting the level of intracellular ara-CTP, and the R-BAC (rituximab, bendamustine, araC) regimen was shown to be effective even in patients resistant to araC thus providing a treatment option even for the elderly and/or frail patients [16,30,31]. It might be speculated that the increased level of ara-CTP might partially offset the anticipated downregulation of DCK thereby explaining, why the combination of bendamustine and araC was shown to be effective even in patients, who relapsed after araC-based therapies [30].

## Conclusions

Our data from the cell lines and primary MCL samples clearly demonstrate that acquired resistance of MCL cells to araC is associated with downregulation of mRNA and protein expression of DCK, enzyme of the nucleotide salvage pathway responsible for phosphorylation of most nucleoside analogs used in anti-cancer therapy. In translation, the results suggest that 1. nucleoside analogs should not be used for the second-line therapy of MCL patients, who fail after araC-based regimen; 2. non-nucleoside analogs should be employed in this setting, including cisplatin, ibrutinib, temsirolimus, bortezomib or bendamustine; 3. ibrutinib appears particularly effective in eliminating araC-resistant MCL cells.

## Methods

### Cell culture

JEKO-1, GRANTA-519 and REC-1 were purchased from German Collection of Microorganisms and Cell Cultures (DSMZ), MINO was from American Tissue Culture Collection (ATCC), HBL-2 was a kind gift of prof. Dreyling (University of Munich, Germany). Cell lines were cultured in Iscove’s modified Dulbecco’s medium (IMDM) supplemented with 15% fetal bovine serum (FBS) and 1% penicillin/streptomycin.

### Reagents

Cytarabine, fludarabine, gemcitabine, cladribine, cyclophosphamide, doxorubicin and cisplatin were from Clinical Dept. of Hematology, University Hospital in Prague, Czech Republic. Temsirolimus, bortezomib, bendamustine and ibrutinib were purchased from Selleck Chemicals. Rituximab was kindly provided by Roche, Czech Republic.

### Establishment of araC-resistant clones

MCL cell lines were incubated in Iscove’s modified Dulbecco’s medium (IMDM) supplemented with 15% fetal bovine serum with increasing concentrations of cytarabine up to 50 μM.

### Proliferation assays

Proliferation was estimated using WST-8 Quick Cell Proliferation Assay Kit (BioVision) according to the manufacturer instructions. Briefly, 5.000 cells were seeded into 96-well plate on day 1. Drugs were added on day 1. Proliferation was measured on day 1 and then since day 4 daily. Antiproliferative activity of each drug was analyzed at several concentrations.

Absorbance of the triplicate samples was measured on ELISA reader after 3 hour incubation with WST-8 reagent at 37 grades Celsius in the thermostat. Maximal absorbance (MAX_u_) obtained from the untreated cells during the particular experiment was arbitrary set as 100%. Absorbance of medium without cells was used as background (B). For each cell population (both, unexposed and drug-exposed) and for each measurement (M_1_, M_2_, M_3_…M_X_) the proliferation curve was calculated as follows: (M_X_ - B)/(MAX_u_ - B). As a consequence, the proliferation curve of untreated cells always peaks 100%, while proliferation curves of drug-exposed cells can terminate below or above 100%.

### ^51^Cr release assay for the assessment of the impact that CD20 mAbs have on rituximab-mediated complement mediated cytotoxicity (CMC) and antibody dependent cellular cytotoxicity (ADCC)

CTRL MCL cells and R clones were labeled with ^51^Cr at 37°C, 5% CO2 for 2 hrs. ^51^Cr-labeled cells were then placed in 96-well plates at a cell concentration of 1 × 10^5^ cells/well (complement-mediated cytotoxicity (CMC) assay) or 1 × 10^4^ cells/well (antibody-dependent cell cytotoxicity (ADCC) assay). Cells were then exposed to rituximab (10 mg/ml) or isotype antibody (10 mg/ml) and human serum (for CMC assay, 1:4 dilution) or peripheral blood mononuclear cells (PBMCs) (for ADCC assay, 40:1 effector: target ratio) for six hrs at 37°C and 5% CO2. ^51^Cr release was measured from the supernatant by standard gamma counting and the percentage of lysis was calculated. PBMCs were obtained from healthy donors (Roswell Park Cancer Institute IRB-approved protocol CIC-016) and isolated by Histopaque-1077 ultracentrifugation of peripheral whole blood and used at an effector: target ratio of 40:1 for ADCC assays. Pooled human serum was used as the source of complement for CMC assays.

### Gene expression profiling and data analysis

A biological duplicate of each araC-resistant MCL clone (R) was compared to a biological duplicate of the original araC-sensitive (CTRL) cell line. In total, five R clones were compared to five corresponding CTRL cell lines using two microarray chips. Total RNA was extracted by RNeasy Mini Kit (Qiagen), and its quality verified using the Agilent 2100 Bioanalyzer (Agilent Technology). Extracted RNA was amplified using the Illumina RNA Amplification Kit (Ambion). Amplified RNA was hybridized to the Illumina HumanRef-8 and HumanRef-12 BeadChips (Illumina). Subsequent data analysis was performed in R-software, mainly in limma package from Bioconductor (http://www.bioconductor.org). Multiple testing correction was performed using Benjamini & Hochberg method. The filtered group of genes with fold change at least ±1.5-fold and adjusted p value < 0.05 were annotated and arranged into biologically relevant categories using The Database for Annotation, Visualization and Integrated Discovery (DAVID, http://david.abcc.ncifcrf.gov).

### Primary MCL sample acquisition, real-time RT-PCR analysis, and apoptosis measurement

All primary MCL samples were obtained from patients with MCL at diagnosis (D1-D10), and at the relapse or during progression after failure of high-dose araC-based front-line therapies (R1-R10). Samples were obtained from patients, who signed informed consent according to the Declaration of Helsinki. Mononuclear cells were isolated from all PBMC and PE samples by the standard Ficoll-Hypaque gradient centrifugation. Mononuclear cells were then CD19 sorted on magnetic columns using CD19 microbeads (Miltenyi Biotec). The purity of MCL population after sorting was > 95% in all cases as verified by flow-cytometry. Total RNA was isolated from CD19-sorted PBMC or PE cells stored in RNA*later* solution using RNeasy Mini Kit (Qiagen, Hilden, Germany) and from fresh-frozen paraffin-embedded (FFPE) lymph node samples using High Pure RNA Paraffin Kit (Roche Diagnostics GmbH, Germany) according to the manufacturer’s instructions. cDNA synthesis was carried out from 1 μg of total RNA with the High-Capacity cDNA Reverse Transcription Kit (random primers) (Applied Biosystems). Real-time RT-PCR was performed using TaqMan Gene Expression Assays on the ABI 7900HT detection system (Applied Biosystems). The reference gene was GAPDH. *Ex vivo* apoptosis of primary MCL cells was determined using Annexin-V-PE (Apronex, Czech Republic) and flow cytometry (BD FACS Canto II) according to the manufacturer’s instructions after 24 hours exposure to 25 μM araC, 100 μM fludarabine and 25 μM gemcitabine.

### Experimental therapy of MCL xenografts

In vivo studies were approved by the institutional Animal Care and Use Committee. Immunodeficient NOD.Cg-*Prkdc*^*scid*^*Il2rg*^*tm1Wjl*^/SzJ mice (Jackson Laboratory) were maintained in individually ventilated cages. JEKO-1 cell line-based mouse model of MCL was used for experiments [32]. JEKO-1 cells were harvested, suspended in PBS, and injected (1 × 10^6^/mouse) i.v. into tail vein of 8- to 12-week-old female mice on DAY 1. Therapy was initiated on DAY 8. Each cohort of mice contained 6–8 animals. The mice received treatment as follows: temsirolimus 1 mg s.c. 1 x weekly (3 cycles), cyclophosphamide 3 mg i.p. 1 × weekly (3 cycles), bendamustine 0.5 mg i.p. two subsequent days (day 1 + day 2) every two weeks (2 cycles), bortezomib 25 μg i.p. 2 × weekly (3 cycles), cisplatin 180 μg i.p. every two weeks (2 cycles), gemcitabine 10 mg i.p. 1 × weekly (3 cycles), fludarabine 1 mg three subsequent days (day 1–3) weekly (3 cycles), rituximab 250 μg s.c. 1 × weekly (3 cycles). The data were analysed in GraphPad Software.

### Two-dimensional electrophoresis

IPG strips (pH 4.0-7.0, 24 cm; ReadyStrip, Bio-Rad) were rehydrated overnight in 450 μL of sample, representing 1.5 mg of protein. Isoelectric focusing was performed for 70 kVh using Protean IEF cell (Bio-Rad). Six replicates were run for each cell type. Focused strips were equilibrated and reduced in equilibration (6 M urea, 50 mM Tris pH 8.8, 30% glycerol, 2% SDS) supplemented with DTT (450 mg per 50 mL) for 15 min and then alkyled in equilibration buffer with added iodacetamide (1.125 mg iodacetamide per 50 mL). SDS-PAGE electrophoresis was performed in a Tris-glycine-SDS system using a 12-gel Protean Dodeca Cell apparatus (Bio-Rad) with buffer circulation and external cooling (20°C). Gels were run at a constant voltage of 80 V per gel for 30 min and then at a constant voltage of 200 V for 6 h. Gels were washed in deionized water to remove redundant SDS and with colloidal Coomassie Brilliant Blue (SimplyBlue™ Safestain, Invitrogen, Carlsbad, CA, USA) overnight.

### Gel image analysis and extraction of peptides

Stained gels were scanned with GS 800 calibrated densitometer (Bio-Rad) and image analysis was performed with Progenesis™ software (Nonlinear Dynamics, Ltd., Newcastle upon Tyne, UK) in semi-manual mode with 6 gel replicates for each cell type. Normalization of gel images was based on total spot density, and integrated spot density values (spot volumes) were then calculated after background subtraction. Average spot volume values (averages from the all 6 gels in the group) for each spot were compared between the groups. Protein spots were considered differentially expressed if their average normalized spot volume difference was > 2-fold. As determined by the Student’s t-test, a p-value < 0.05 was considered to indicate a statistically significant difference.

### Protein digestion and peptide extraction

Spots containing differentially expressed proteins were excised from the gels, cut into small pieces and washed 3 times with 25 mM ammonium bicarbonate in 50% acetonitrile (ACN). The gels were then dried in a SpeedVac Concentrator (Eppendorf, Hamburg, Germany). Sequencing grade modified trypsin (Promega, Madison, WI, USA) (6 ng/μl in 25 mM ammonium bicarbonate in 5% ACN) was added. Following overnight incubation at 37°C, the resulting peptides were extracted with 50% ACN.

### MS analysis and protein identification

Peptide samples were spotted on a steel target plate (Bruker Daltonics, Bremen, Germany) and allowed to dry at room temperature. Matrix solution (3 mg α-cyano-4-hydroxycinnamic acid in 1 ml of 50% ACN containing 0.1% trifluoroacetic acid) was then added. MS was performed on an Autoflex II MALDI-TOF/TOF mass spectrometer (Bruker Daltonics, Bremen, Germany) using a solid nitrogen laser (337 nm) and FlexControl software in reflectron mode with positive ion mass spectra detection. The mass spectrometer was externally calibrated with Peptide Calibration Standard II (Bruker Daltonics). Spectra were acquired in the mass range 800–3,000 Da. The peak lists were generated using FlexAnalysis and searched against Swiss-Prot (2012_07 version, 536 789 sequences) using Mascot software. The peptide mass tolerance was set to 100 ppm, taxonomy Homo sapiens, missed cleavage was set to 1, fixed modification for cysteine carbamidomethylation, and variable modifications for methionine oxidation and protein N-terminal acetylation. Proteins with Mascot score over the threshold 56 for *p <* 0.05 calculated for the used settings were considered as identified. If the score was lower, the identity of protein candidate was confirmed by MS/MS.

### Western blot analysis

Cells were lysed in NHT buffer (140 mM NaCl, 10 mM HEPES, 1.5% Triton X-100, pH 7.4). Protein concentration in the collected supernatants was determined by the Bradford assay (Bio-Rad). Lysate samples (50 μg) were combined with SDS loading buffer containing 2-mercaptoethanol and boiled for 5 min. Quadruplicate samples were separated on 12% SDS-PAGE minigels in Tris-glycine buffer (Bio-Rad). Electrophoresis was performed at a constant voltage for 30 min at 45 V per gel, and then at 90 V per gel until the dye front reached the gel bottom. Proteins were transferred onto 0.45 μm PVDF membranes (Milipore, Billerica, MA, USA) in a semi-dry blotter (Hoefer, San Francisco, CA, USA) at 0.8 mA/cm^2^. Membranes were incubated in PBS (Invitrogen) containing 0.1% Tween-20 and 5% non-fat dried milk for 1 h. GAPDH or Actin were used as the loading controls. As primary antibodies anti-deoxycytidine kinase mouse monoclonal antibody (sc 81245 Santa Cruz Biotechnology, Sanat Cruz, CA, USA) diluted 1:200 or polyclonal anti-GAPDH produced in rabbit (Sigma-Aldrich, G9545) diluted 1:10,000 were used. After thorough washing in blocking buffer, a secondary horseradish peroxidase-conjugated anti-mouse (sc2005) or anti-rabbit antibody (sc2313) (both from Santa Cruz Biotechnology) was added (1:10,000). The signal was detected using LumiGLO Reserve, (KPL, Gaithersburg, MD, USA) or Western Blotting Luminol Reagent (Santa Cruz Biotechnology, Inc., Santa Cruz, CA, USA) and membranes were exposed to X-ray films (Kodak, Rochester, NY, USA).

## Abbreviations

ACN: Acetonitrile; ADCC: Antibody-dependent cytotoxicity; AML: Acute myelogeneous leukemia; BTK: Bruton tyrosine-kinase; araC: Cytarabine, a pyrimidine analog used for anticancer therapy; CDA: Cytidine-deaminase, a key inactivating enzyme of nucleotide salvage pathway; CMC: Complement-mediated cototoxicity; CTRL: araC-sensitive MCL cell line; DCK: Deoxycytidine-kinase, a rate-limiting enzyme of nucleotide salvage pathway; DTT: Dithiothreitol; FFPE: Fresh-frozen paraffin-embedded (lymph node sections); HDAC: High-dose araC; i.v.: Intravenous injection; MCL: Mantle cell lymphoma; NHL: Non-Hodgkin lymphoma; NT5C2: Cytoplasmic 5′nucleotidase II, a key inactivating enzyme of nucleotide salvage pathway; PBMC: Peripheral-blood mononuclear cells; PE: Pleural effusion; R: araC-resistant clone derived from araC-sensitive cell line; R-CHOP: A combination of rituximab, cyclophosphamide, doxorubicin, vincristin and prednisone; R-DHAP: A combination of rituximab, high-dose cytarabine, cisplatin and dexamethasone; RR-MCL: Relapsed/refractory mantle cell lymphoma; s.c.: subcutanous injection.

## Competing interests

The authors declare that they have no competing interests.

## Authors’ contributions

PK and JP conceived of the study and participated in drafting of the manuscript. MK carried out gene expression analysis and *in vivo* experiments, OV, LL and JP carried out proteomic analysis and western blotting. BM, DV, JM, PV and LL participated in *in vitro* experiments. CM carried out chrome-releasing assays. VK performed the statistical analysis. FH and MV participated in the design of the study and helped to review the manuscript. RJ and MT carried out analysis of primary MCL samples. All authors read and approved the final manuscript.

## Authors’ informations

Jiri Petrak and Pavel Klener Jr are considered senior co-authors.

## Supplementary Material

Additional file 1: Figure S1 Functional cathegories of genes differentially expressed in R compared to CTRL as determined by DAVID. The filtered group of genes acquired from all five MCL cell lines with fold change at least ±1.5-fold and adjusted p value < 0.05 were annotated and arranged into biologically relevant categories using The Database for Annotation, Visualization and Integrated Discovery (DAVID, http://david.abcc.ncifcrf.gov). Click here for file

Additional file 2: Table S1 List of genes differentially expressed in more than one R clone compared to the corresponding CTRL cells. Microarray data are shown in Additional file 2: Table S1. 31 genes were differentially expressed in two araC-resistant clones (R) compared to the corresponding araC-sensitive controls (CTRL). 1 gene (TPM1) was differentially expressed in three R clones, and 1 gene (DCK) was differentially expressed in all five R clones compared to the corresponding CTRL. Click here for file

Additional file 3: Figure S2 Ibrutinib appears more cytotoxic to cytarabine-resistant (R) compared to cytarabine-sensitive (CTRL) MCL cells. WST-8 cell proliferation assays of CTRL cells and R clones were carried out as described in Methods. Maximal absorbance obtained from the untreated cells during the particular experiment (MAX_u_) was arbitrary set as 100%. Absorbance of medium without cells was used as background (B). For each cell population (both, unexposed and drug-exposed) and for each measurement (M_1_, M_2_, M_3_…M_X_) the proliferation curve was calculated as follows: (M_X_ - B)/(MAX_u_ - B). As a consequence, proliferation curves of untreated cells always peak at 100%, while proliferation curves of drug-exposed cells can terminate below or above 100%. One representative example of two independent experiments carried out on REC-1, HBL-2 and GRANTA-519 is shown. In summary, REC-1 R clone was > 100-fold sensitive to Bruton tyrosine-kinase (BTK) inhibitor ibrutinib compared to REC-1 CTRL cells. Both HBL-2 and GRANTA-519 R clones were approx. 2-fold more sensitive to ibrutinib compared to HBL-2 and GRANTA-519 CTRL cells. Click here for file
