# Supplementary figures and images for: Downregulation of deoxycytidine kinase in cytarabine-resistant mantle cell lymphoma cells confers cross-resistance to nucleoside analogs gemcitabine, fludarabine and cladribine, but not to other classes of anti-lymphoma agents
Source: Mol Cancer. 2014 Jun 27;13:159. doi: 10.1186/1476-4598-13-159 (PMC4094598; doi:10.1186/1476-4598-13-159)

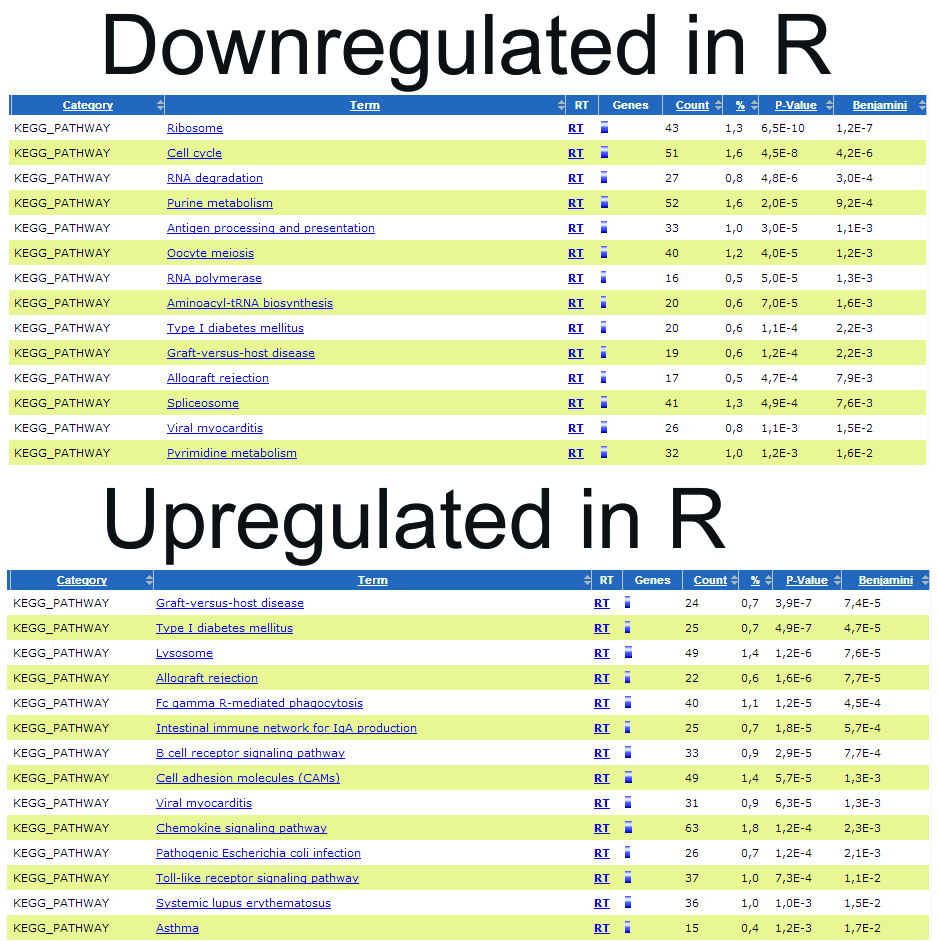

Supplement: Additional file 1: Figure S1 — Functional cathegories of genes differentially expressed in R compared to CTRL as determined by DAVID. The filtered group of genes acquired from all five MCL cell lines with fold change at least ±1.5-fold and adjusted p value < 0.05 were annotated and arranged into biologically relevant categories using The Database for Annotation, Visualization and Integrated Discovery (DAVID, http://david.abcc.ncifcrf.gov). [file 1476-4598-13-159-S1.jpeg]

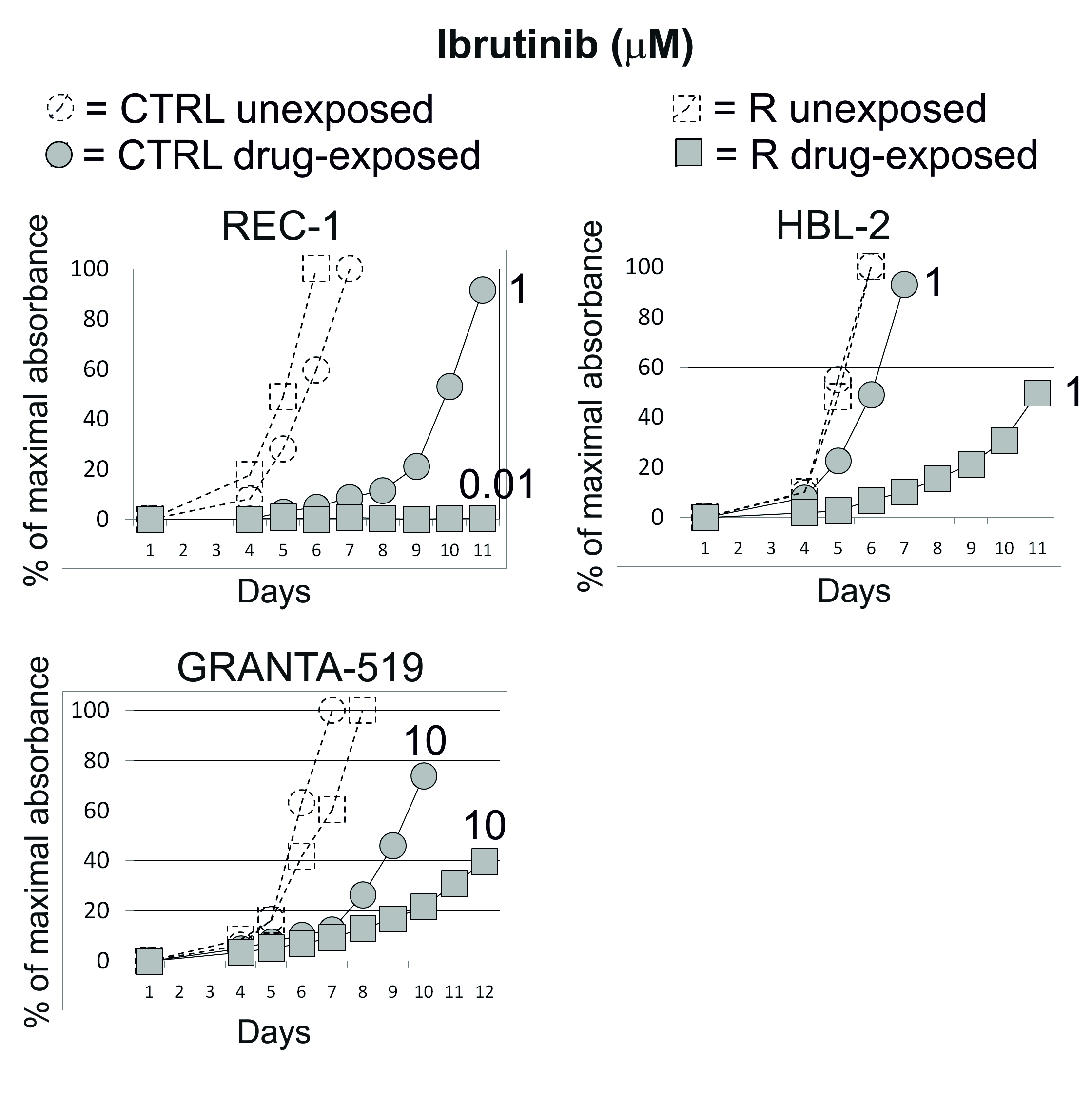

Supplement: Additional file 3: Figure S2 — Ibrutinib appears more cytotoxic to cytarabine-resistant (R) compared to cytarabine-sensitive (CTRL) MCL cells. WST-8 cell proliferation assays of CTRL cells and R clones were carried out as described in Methods. Maximal absorbance obtained from the untreated cells during the particular experiment (MAXu) was arbitrary set as 100%. Absorbance of medium without cells was used as background (B). For each cell population (both, unexposed and drug-exposed) and for each measurement (M1, M2, M3…MX) the proliferation curve was calculated as follows: (MX - B)/(MAXu - B). As a consequence, proliferation curves of untreated cells always peak at 100%, while proliferation curves of drug-exposed cells can terminate below or above 100%. One representative example of two independent experiments carried out on REC-1, HBL-2 and GRANTA-519 is shown. In summary, REC-1 R clone was > 100-fold sensitive to Bruton tyrosine-kinase (BTK) inhibitor ibrutinib compared to REC-1 CTRL cells. Both HBL-2 and GRANTA-519 R clones were approx. 2-fold more sensitive to ibrutinib compared to HBL-2 and GRANTA-519 CTRL cells. [file 1476-4598-13-159-S3.jpeg]
